# Abobotulinum - a toxin injection in patients with refractory idiopathic detrusor overactivity: injections in detrusor, trigone and bladder neck or prostatic urethra, versus detrusor - only injections

**DOI:** 10.1590/S1677-5538.IBJU.2016.0622

**Published:** 2017

**Authors:** Maryam Emami, Pejman Shadpour, Amir H. Kashi, Masoud Choopani, Mohammadreza Zeighami

**Affiliations:** 1 Hasheminejad Kidney Center (HKC), Iran University of Medical Sciences (IUMS), Tehran, Iran

**Keywords:** Urinary Bladder, Overactive, Botulinum Toxins

## Abstract

**Purpose:**

To evaluate if the injections of abobotulinum-A toxin in trigone and bladder neck/prostatic urethra in addition to detrusor provides better symptoms relief and urodynamic findings in patients with idiopathic detrusor overactivity (IDO) refractory to medical treatment.

**Materials and Methods:**

A total of 74 patients with IDO refractory to anticholinergics received injections in detrusor, trigone and bladder neck/prostatic urethra (Group A, N=36) versus detrusor only injections (Group B, N=38) of abobotulinum-A toxin. All patients were evaluated by a standard overactive bladder symptom score (OABSS) questionnaire and cystometrography before and 6 weeks after the operation. OABSS questionnaire was also completed 20 weeks after the operation.

**Results:**

The magnitude of OABSS reduction from baseline to 6 weeks after operation in groups A and B patients was 13.4±2.2 versus 11.7±2.1 (p=0.001). Cystometry results were similar in both groups except for higher volume at urgent desire to void in Group B patients (p <0.001). The mean±SD change in residual volume in Group A at 6 weeks after the operation was −4.8±28.6mL (p=0.33) compared to 21.3±16.9mL in Group B patients (p <0.001).

**Conclusions:**

In patients with IDO, adding trigone, and bladder neck/prostatic urethra as sites of abobotulinum- A toxin injection produces greater reductions in OABSS score and less residual urine volume but a lower volume at urgent desire to void in comparison with detrusor only injections.

## INTRODUCTION

Overactive bladder is defined as lower urinary tract symptoms including urgency with or without urge incontinence, sometimes accompanied by nocturia and frequency (1). It affects about 17% of adult European population (2). In patients with idiopathic detrusor overactivity refractory to anticholinergic therapy, intravesical injection of botulinum toxin has emerged as a second line minimally invasive treatment (3, 4).

Botulinium toxin A (BoNTA) is a potent neurotoxin produced by Clostridium botulinum (5). The two commonly used products in urology are Botox (Onabotulinum toxin A) and Dysport (Abobotulinum toxin A). BoNTA is an effective treatment in patients with idiopathic overactive bladder.

Currently there is no consensus about the exact dose or sites for injection of this toxin (6). The trigone had been spared of BoNTA injection fearing the theoretical risk of vesicoureteral re-flux. However, Karsenty et al. and Mascarenhas et al. reported that trigonal injection of BoNTA will not induce reflux in patients with overactive bladder (7, 8). The first prospective randomized controlled trial by Abdel-Meguid displayed the superiority of trigonal BoNTA injection in neurogenic bladder patients over trigonal sparing injections (9). A randomized clinical trial by Manchesha et al. also pointed to the superiority of trigone including injections for IDO patients, but this study included few patients.

A recent meta-analysis on six studies by Davis et al. revealed that there is no significant difference between trigonal and extra-trigonal BoNTA injections in terms of adverse effects and short term efficacy and that more trials are needed to define the optimal injection techniques and sites for delivery of intravesical BoNTA (6).

The primary objective of this study was to evaluate the efficacy of BoNTA injections in detrusor, trigone, and bladder neck/prostatic urethra in comparison with detrusor only injections. Patient satisfaction and urodynamic findings in follow-up were used to assess the efficacy of injections. We introduced new sites of injection around bladder neck in women and prostatic urethra in men and evaluated them in distinction to the current literature.

## MATERIALS AND METHODS

### Studied population

This prospective study was performed between April 2012 and July 2015. Patients older than 18 years old with IDO proven by urodynamic study, refractory to anticholinergic therapy (for at least 3 months) were recruited. Neurology consult was requested in young patients and subjects with atypical clinical presentation or severe contractions on cystometrogram. Patients were excluded in case of coagulopathy or neurological detrusor overactivity. Patients were also excluded if they had history of previous BoNTA injection or surgery of the genitourinary tract. Urinalysis and culture were performed to rule out patients with urinary tract infection prior to surgery. Male patients with clinical or urodynamic evidence of bladder outlet obstruction were excluded from the study.

All patients underwent voiding cystourethrography (VCUG) before surgery and reflux was not detected in any of them. Anticholinergic therapy was discontinued in all patients 7 days prior to injection.

### Baseline Assessment

All patients were assessed at baseline by history, physical examination, the Overactive Bladder Symptom Score (OABSS) questionnaire, and urodynamic examination. Symptoms were evaluated according to the validated seven questions of OABSS questionnaire (score range: 0-28) (10). Studied urodynamic parameters consisted of maximum cystometric capacity (MCC), maximum detrusor pressure in filling phase (MDP), volume at first desire to void (VFDV), and volume at urgent desire to void (VUDV). Post void residue (PVR) was also assessed by abdominal ultrasonography.

### Injection technique

**Figure 1:**
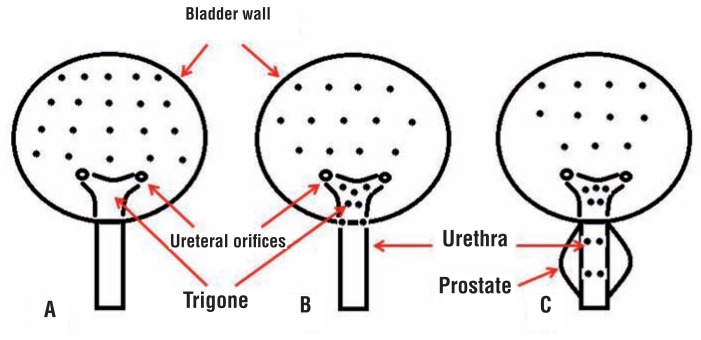
schematic diagram for sites of botulinum toxin A injection: A) Group B patients, male or female (20 detrusor only injections); B) Group A patients, female (13 injections into detrusor, 5 trigone injection and 2 bladder neck injections at 5 and 7 o'clock positions); c) Group A patients, male (11 detrusor injection, 5 trigone injections, and 4 injections in the proximal and distal prostatic urethra at 3 and 9 o'clock positions).

Patients received deep IV sedation, and then spinal anesthesia. We have performed the last 8 injections under sedation. 200mg IV ciprofloxacin was given to all patients 1/2 hour before the operation. All injections were performed by an expert female urologist. We used 500U BoNTA and 20 injection sites in each patient. Botulinium toxin A (Dysport) (500U) was reconstituted with 20cc saline 0.9%. Cystoscopy was performed with 21Fr rigid cystoscope in lithotomy position and after filling bladder with 150mL of irrigation fluid, intradetrusor injections were performed by a 27G disposable needle. Choice of injection into trigone and bladder neck/prostatic urethra versus detrusor only injections was made at the discretion of the operating surgeon. Generally, patients with storage predominant symptoms were more likely to be included in the detrusor only group (Group B) and patients with emptying predominant symptoms were more likely to be included in the detrusor, trigone, and bladder neck/prostatic urethra group (Group A). Group A patients received 2 injections in the bladder neck in female patients and 4 injections in male patients in proximal and distal prostatic urethra as illustrated in Figure-1, the remaining sites included 11 or 13 injections into detrusor and 5 trigonal injections away from ureteral orifices. Patients with detrusor only injections (Group B) received 20 injections of the same preparation at different parts of detrusor excluding trigone (Figure-1). The depth of injection in detrusor, trigone and bladder neck was about 2mm as estimated by insertion of half of the 4-mm needle. Bladder neck injections in female patients were performed at 5 and 7 o'clock positions (Figure-1). In male patients, prostatic urethra injections were performed in the proximal and distal prostatic urethra at 3 and 9 o'clock positions (Figure-1) with the injection needle fully inserted into the prostatic tissue (4mm depth of injection).

### Follow-up

Foley catheter was discontinued the day after surgery. Urinalysis and urine culture were requested two weeks after treatment and in this clinic visit PVR was assessed by urethral catheter. If PVR was less than 100mL, patients were allowed to continue anticholinergic medications with half of the original pre-treatment dose. Patients were re-evaluated at 6 and 20 weeks after treatment by history, physical examination, and OABSS questionnaire. Urodynamic study and PVR were reevaluated at 6 weeks after injections.

#### Statistical analysis

Student t-test was used to compare quantitative variables at baseline and also for comparing the magnitude of difference from baseline to follow-up visits between treatment groups. Fisher exact or chi-square tests were used to compare nominal data at baseline. Paired t-tests were used to compare follow-up data with their baseline values.

The ethics of this study was approved by the Hasheminejad Kidney Center ethics committee and are in accordance with the Helsinki declaration of 1964 and its later amendments. Patients were explained about the study objectives in their own language. Written informed consent was obtained from all patients.

## RESULTS

A total of 74 patients (38 males and 36 females) aged 21 to 59 years (mean±SD: 39.1±14.0) were studied. Thirty-six patients were included in Group A (18 females and 18 males) and 38 in Group B (18 females and 20 males). The mean±SD of patients' age in Groups A and B was 39.1±14.4 and 39.0±14.0. Table-1 summarizes OABSS scores at baseline and at 6 and 20 weeks after injections.

**Table 1:** Overactive Bladder Symptom Scores (OABss) at baseline and at 6 & 20 weeks after Abobotulinum toxin injections.

		Group A*	Group B**	P-value
Number of patients		36	38	
Age, years; mean±SD		39.1±14.4	39.0±14.0	0.99
**Total OABSS**				
	Baseline, mean (range)	22.3 (17–28)	21.8 (18–26)	0.41
	6 weeks, mean (range)	8.9 (–14)	10.1 (8–14)	0.006
	Compared to baseline (P value)	<0.001	<0.001	
	20 weeks, mean (range)	10.9 (8–17)	13.4 (9–17)	<0.001
	Compared to baseline (P value)	<0.001	<0.001	

* Detrusor, trigone, bladder neck/prostatic urethra including injections

** Detrusor only injections

**OABSS =** Overactive Bladder Symptom Score

The magnitudes of reduction in OABSS score from baseline to 6 weeks after injections in Groups A versus B were 13.4±2.2 versus 11.7±2.1 (p=0.001). The magnitudes of reduction in OABSS score from baseline to 20 weeks after injections in Groups A versus B were 11.4±2.2 versus 8.3±2.2. (p<0.001) The magnitude of reduction in OABSS score in both treatment groups was not related to gender or age of patients. Regarding PVR, no statistically significant change in PVR was observed in Group A patients relative to baseline while statistically significant increase in PVR was observed in Group B patients. The change in PVR was dependent on gender of patients. The mean±SD of PVR increase in male patients was 0.1±31.4mL versus 17.6±16.6mL for female patients. (p=0.004) This difference remained statistically significant after controlling for treatment Groups of A and B.

One patient developed urinary retention in each group and responded to clean intermittent catheterization.

Regarding urodynamic parameters, apart from VUDV other urodynamic parameters did not achieve statistically significant difference between the treatment groups (Table-2).

**Table 2:** Cystometric parameters at baseline and at 6 weeks after Abobotulinum toxin injections.

		Group A*	Group B**	P-Value
**MCC, mL; Mean (range)**				
	Before injection	224.44 (160–400)	284.59 (160–520)	0.61
	6 week after injection	385.69 (280–490)	409.82 (290–530)	0.11
	P-value (6 week to baseline)***	0.08	0.07	
**MDP, cm H_2_O; Mean (range)**				
	Before injection	39.9 (14–63)	40.56 (17–73)	0.86
	6 week after injection	14.75 (6–29)	16.3 (7–30)	0.53
	P-value (6 week to baseline)***	0.8	0.9	
**VUDV, mL; Mean (range)**				
	Before injection	211.45 (110–360)	205.68 (134–310)	0.73
	6 week after injection	334.13 (200–456)	363.93 (254–476)	0.02
	P-value (6 week to baseline)***	<0.001	<0.001	
**VFUV, mL; Mean (range)**				
	Before injection	175.59 (100–330)	164.21 (110–290)	0.49
	6 week after injection	288.06 (200–450)	298.24(200–404)	0.32
	P-value (6 week to baseline)***	0.1	0.08	
**PVR, mL; Mean (range)**				
	Before injection	64.6 (20–140)	69.3 (20–170)	0.61
	6 week after injection	60.8 (43–920)	90.6 (44–190)	<0.001
	P-value(6 week to baseline)***	0.33	<0.001	

* Detrusor, trigone, bladder neck/prostatic urethra including injections

** Detrusor only injections

*** Within Group p-value: Comparison cyctometric varaibles 6 weeks after injections with values before injection

**PVR =** Post void residue; **MCC =** Maximum cystometic capacity; **MDP =** Maximum detrusor pressure in flling phase; **VFUV =** Volume at first desire to void; **VUDV =** Volume at urgent desire to void

8 patients in Group A and 10 patients in Group B needed a reinjection of BoNTA injection after follow-up at 20 weeks (p=0.77).

## DISCUSSION

The efficacy of intravesical BoNTA as an alternative therapy for bladder overactivity refractory to anticholinergics has been proven (11, 12). Denys et al. in a double blind, dose rating, placebo-controlled study with 6 month follow—up observed >50% improvement in UUI and urgency in 65% and 56% of patients who had received 100U and 150U of BoNTA (13). Kuo did not detect any difference in outcome relative to the injecting dose (14). In a randomized clinical trial, Manecksha et al. reported on the superiority of detrusor+trigonal injections of BoNTA in terms of reduction in OABSS score in comparison with detrusor only injections (15).

A problem with injections of BoNTA was the increase in residual urine volume which can potentially predispose to UTI (14, 16, 17). Some studies reported that patients with idiopathic detrusor overactivity (IDO) contract their bladder neck to prevent urinary incontinence. So that, during voiding funneling of bladder neck is incomplete and this results in high residual volume in these patients. Abdel-Meguid et al. reported >40% reduction in maximal urinary flow rate after injection of BoNTA (9) leaving patients with bladder outlet obstruction in state of high residual urine volume. Previous reports point to the positive effects of alpha adrenergic blocking agents in improving lower urinary tract symptoms and residual urine volume in female and male patients with lower urinary tract symptoms (18, 19). Therefore, we hypothesized that injection of BoNTA into bladder neck and prostatic urethra can further improve the symptoms and probably residual urine volume. Consequently, we introduced new sites for injection at bladder neck and prostatic urethra to address these issues in our study.

The results of our study show that Group A had a lower residual urine volume in comparison with Group B patients. Thus, we expect older patients with large PVR and a greater risk of acute urinary retention, to be fit for the effects of bladder base BoNTA injection. It is established that large PVR volume especially in elderly patients is associated with repeated episodes of UTI. This injection site is especially important in male patients in whom bladder neck has an important resistive role in the outflow of urine.

It has been demonstrated that BoNTA affects the afferent nerve endings and consequently influences symptoms especially frequency and urgency, as studied by Schemid et al. (20). High concentration of nerve endings in the trigonal area indicates that injection of BoNTA in this area may ameliorate patient symptoms as shown by our study. Patients in Group A showed lower mean OABSS compared to Group B at 6 weeks after treatment and this difference remained statistically significant 20 weeks post treatment.

We observed a low rate of retention after the procedures (2 patients; 3%). Overall retention rate after the study by Manchesha et al. was 9% (14) while in the study by Kuo et al., this rate was near 6% (19). The type, brand, dose and injection sites of BoNTA injection have been proposed as possible reasons for observation of different adverse effects/efficacy endpoints (18). Furthermore, we inserted overnight urinary catheter which was performed in Kuo et al. study with lower retention rates but not in the study by Manchesha et al. with higher retention rates.

Nevertheless, there are studies which reported no superiority of detrusor plus trigonal injections in comparison with detrusor only injections. Lucioni et al. used onabotulinum toxin A with two injections in trigone. They reported no difference in the trigonal group compared with detrusor group (6). Kuo et al. used 100U onabatulinum toxin A with 10 injection sites in the bladder base in one group and 20 injection sites in bladder body or body and trigone and reported comparable outcomes (19). Part of this inconsistency can be explained by difference in total injected doses of BoNTA or its formulations as indicated by Davis et al. in a recent meta-analysis (18).

Urodynamic parameters in Groups A and B were similar at baseline and follow-up urodynamic studies including MCC, VFDV, and MDP except for a higher VUDV in Group B patients 6 weeks after injections. Manecksha et al. randomized patients to trigone-spared group with 20 sites of injection into the bladder wall, and trigone-including group with 5 injections into the trigone and 15 injections into the bladder wall. No obvious between groups difference was shown in VUDV at 6 weeks postoperative assessment (14).

We observed minor complications such as slight hematuria that stopped spontaneously 24 hour after procedure. Acute urinary retention was observed only in one patient in each group which responded to temporary catheterization. Antibiotic therapy was continued up to 1 week after injections. During further follow-up none of our patients developed UTI.

There are some limitations in our study. This study was not a randomized study. Therefore, the possibility of bias in patient selection cannot be ruled out. All cystoscopies were carried out by rigid cystos-cope. Using flexible cystoscopies can reduce trauma and will lead to a more tolerable cystoscopic procedure with light anesthesia.

## CONCLUSIONS

Adding trigone, and bladder neck or prostatic urethra as sites of BoNTA injection in patients with IDO produces greater reductions in OABSS score and less residual urine volume but a lower volume at urgent desire to void in comparison with detrusor only injections.
